# Using Machine-Learning to Predict Sleep-Disordered Breathing Diagnosis From Medical Comorbidities and Craniofacial Features

**DOI:** 10.7759/cureus.39798

**Published:** 2023-05-31

**Authors:** Stephen Cokim, Joshua Ghaly

**Affiliations:** 1 Medicine, Oceania University of Medicine, Apia, WSM; 2 Internal Medicine, Mackay Base Hospital, Mackay, AUS

**Keywords:** machine learning, craniofacial, obstructive sleep apnoea, sleep disordered breathing, sleep apnoea syndromes

## Abstract

Objectives

This paper attempts to use machine-learning (ML) algorithms to predict the presence of sleep-disordered breathing (SDB) in a patient based on their body habitus, craniofacial anatomy, and social history.

Materials and methods

Data from a group of 69 adult patients who attended a dental clinic for oral surgeries and dental procedures in the last 10 years was used to train machine-learning models to predict whether a subject is likely to have SDB based on input information such as age, gender, smoking history, body mass index (BMI), oropharyngeal airway (Mallampati assessment), forward head posture (FHP), facial skeletal pattern, and sleep quality.

Logistic Regression (LR), K-nearest Neighbours (kNN), Support Vector Machine (SVM) and Naïve Bayes (NB) were selected as these are the most frequently used supervised machine-learning models for classification of outcomes. The data was split into two sets for machine training (80% of total records) and the remaining was used for testing (validation).

Results

Initial analysis of collected data showed overweight BMI (at 25 or above), periorbital hyperchromia (dark circle eyes), nasal deviation, micrognathia, convex facial skeletal pattern (class 2) and Mallampati class 2 or above have positive correlations with SDB.

Logistic Regression was found to be the best performer amongst the four models used with an accuracy of 86%, F1 score of 88% and area under the ROC curve (AUC) of 93%. LR also had 100% specificity and 77.8% sensitivity. Support Vector Machine was the second-best performer with an accuracy of 79%, F1 score of 82% and AUC of 93%. k-Nearest Neighbours and Naïve Bayes performed reasonably well with F1 scores of 71% and 67%, respectively.

Conclusions

This study demonstrated the feasibility of using simple machine-learning models as a credible predictor of sleep-disordered breathing in patients with structural risk factors for sleep apnoea such as craniofacial anomalies, neck posture and soft tissue airway obstruction. By utilising higher-level machine-learning algorithms, it is possible to incorporate a broader range of risk factors, including non-structural features like respiratory diseases, asthma, medication use, and more, into the prediction model.

## Introduction

Sleep-disordered breathing (SDB) is the most common, underdiagnosed sleeping disorder in the general population. It is estimated that 25% of adults have mild SDB [1]. SDB refers to any condition where breathing is disturbed or impaired during sleep. SDB covers not only primary snoring, catathrenia, sleep-related hypoventilation, and sleep-related hypoxemia but also a spectrum of sleep apnoea including obstructive sleep apnoea (OSA), central sleep apnoea and mixed sleep apnoea [2].

There are anatomical, neurologic, and physiologic factors which contribute to SDB. Structural causes include large neck circumference, obesity, narrow mandible, retrognathia, dental malocclusion, overbite, reduced nasal patency, high and narrow hard palate, elongated and low-lying uvula, enlarged tonsils and adenoids, macroglossia. Some of these features individually are benign. However, on the whole, they all contribute to restricted air intake during sleep [3,4]. Non-structural risk factors for SDB are male sex, age, postmenopausal state, alcohol or sedative use, smoking, snoring, and sleep position [5].

The diagnosis of SDB would often require a series of questionnaires and a polysomnography (PSG) as the gold standard. PSG, however, is cumbersome, inconvenient, time-consuming, and expensive, requiring specialists to administer and interpret. More importantly, the test is inherently intrusive which does not reflect the patient’s true natural depth of sleep. For these reasons, PSG is underutilised resulting in a large number of undiagnosed SDB [6].

In this paper, we explore the possibility of using basic machine-learning models as an adjunct screening or diagnostic tool for clinicians to predict SDB using data collected from routine clinical examinations in the office without a reliance on PSG. Four models selected for this study are Logistic Regression (LR), Support Vector Machine (SVM), K-Nearest Neighbour (kNN) and Naïve Bayes (NB). They were selected because they are most frequently used for simple classification of whether an outcome is positive or negative [7,8], which in this case, is a prediction of whether SDB is present or not.

## Materials and methods

In this cross-sectional retrospective study, we conducted a chart review of patients who attended a private dental clinic in Brisbane over a 10-year period (2007-2017) for complex dental procedures or craniofacial adjustments that required extended management, including cone-beam computed tomography (CBCT) imaging and multiple rounds of interventions, by an oral surgeon.

We searched the medical records for patients who have at least one of the physical or craniofacial features that suggest they may have a reduced pharyngeal airway and, thus, a greater risk of having SDB. The features of interest (Table 1) were BMI, smoking history, neck circumference, lip incompetence, micrognathia, retrognathia, dentofacial skeletal pattern, periorbital hyperchromia, and Mallampati grading. Any symptoms of sleep disorders, such as self-reported complaints of snoring, fragmented sleep, or daytime sleepiness, were recorded. Any patient who complains of a sleeping disorder but does not exhibit any of the associated clinical features mentioned above was also included in this study.

**Table 1:** Features and target of sample data collected for machine-learning. *Craniofacial features and Mallampati grading were performed by an experienced oral surgeon with accreditation in Conscious Sedation.

Features (model input)	
	Patient ID (numeric integer)
	Gender (0 = female, 1 = male)
	Age (numeric integer)
	Neck Circumference (43cm for Males, 40cm for Females) (0 = less than, 1 = more than)
	BMI (0 = less than 25, 1 = greater than or equal 25)
	Smoking habit (1 = no, 2 = yes, 3 = ex-smoker)
	Forward Head Posture (1 = no, 2 = yes)*
	Lip incompetence (1 = no, 2 = yes)*
	Dark circle eyes (1 = no, 2 = yes)
	Nasal Deviation (1 = no, 2 = yes)*
	Skeletal Pattern (class 1 2 3)
	Micrognathia (1 = no, 2 = yes)*
	Mallampati classification (1,2,3,4)*
Target (model prediction)	
	SDB features - indicative of SDB (0 = no, 1 = yes)

Overall, we collected a sample of 69 subjects. Thirty-eight patients have self-reported complaints of snoring, fragmented sleeps, or daytime sleepiness. These patients are considered to have a high likelihood of SDB. The remaining 31 patients denied having any sleeping difficulties but have at least one anatomical or clinical feature listed in Table 1.

All participants consented to the collection and release of their medical information for this study which includes age, gender, social history, body habitus, craniofacial features, and sleep satisfaction. Informed consents were obtained and received human ethics approval from Griffith University, Queensland, Australia (Ref No: 2017/626).

Data preparation

The data obtained was distilled into machine-learning formats of Features and Test. The train-test split method was then used to randomly allocate 55 records from the dataset to the training set and the remaining 20% (14 records) is used as a test set for ML to compare its predictions against.

Machine-Learning software

Data was trained, analysed, and plotted on Kaggle using Pandas, Numpy, SKLearn and Matplotlib libraries of Python version 3.11.3.

## Results

The summary statistics of the data collected are shown in Table 2. Out of the sample size of n=69, 38 participants (55%) are deemed to have SDB based on clinical findings, CBCT imaging and/or patient’s own assessment. This is considered as a good, well-balanced, low-biased dataset for machine learning because SDB is the test dataset in which the models are learned and validated against. If there are too many of either positive or negative SDB subjects in the data, then the models will eventually train towards those results.

**Table 2:** Summary snapshot of sampled data consisting of 69 samples with 38 deemed as having sleep-disordered breathing (SDB). FHP: Forward head posture

Total number of participants, n=69	Frequency	% of all samples
Male	18	26%
BMI>=25	25	36%
FHP	56	81%
Periorbital hyperchromia (dark circle eyes)	52	75%
Nasal Deviation	47	68%
Micrognathia	16	23%
Never smoked	60	87%
Current Smoker	5	7%
Ex-smoker	4	6%
Mallampati class 1	27	39%
Mallampati class 2	28	41%
Mallampati class 3	13	19%
Mallampati class 4	1	1%
Skeletal Pattern class 1	36	52%
Skeletal Pattern class 2	27	39%
Skeletal Pattern class 3	6	9%
SDB features	38	55%

The average age of participants was 62. If we consider underweight representation as less than 40% of the sample size, male gender (26%), BMI>=25 (36%), micrognathia (23%), previous or current smoker (13%), normal Mallampati (39%), and convex skeletal pattern class 2 (39%) were the underrepresented features found in this study. On the flip side, never smoked (87%), Mallampati class 2 or above (61%), periorbital hyperchromia (75%) and forward head posture (81%) were overrepresented in this dataset.

After the models have been trained, the resulting performance metrics (Table 3) suggest all models performed well. Logistic Regression was the best performer, standing out from others with the highest F1 score, that is, the lowest false positives and lowest false negatives. Support Vector Machine also performed well with the second highest F1 score. Logistic Regression and Support Vector Machine have the same highest scores in Area under the ROC curve (AUC). Recall is an important metric in disease detection because we want to minimise false negatives with a high recall score. LR and SVM both have the same high recall (0.78), whilst both kNN and NB lag. Naïve Bayes was the worst performer in the test with the lowest F1 score and sensitivity.

**Table 3:** Performance comparison of machine-learning (ML) models in predicting sleep-disordered breathing (SDB). Accuracy is the number of times the model made a correct prediction across the entire dataset. Lower and Upper Confidence Interval (CI) of Accuracy scores are at 95% interval. Accuracy was widely used during the initial introduction of machine learning. It is now superseded by F1 score which combines Precision (Positive Predictive Value) and Recall (Sensitivity) into a single metric. F1 score = (2 x Precision x Recall)/(Precision + Recall). The above scores are expressed as ratio except for Specificity and Sensitivity which are percentages.

Model	Accuracy	Lower CI	Upper CI	Precision	Recall	F1 score	Specificity	Sensitivity	Area under curve
Logistic Regression	0.86	0.67	1.04	1.00	0.78	0.88	100.0	77.8	0.93
k-Nearest Neighbours	0.71	0.48	0.95	1.00	0.56	0.71	100.0	55.6	0.89
Support Vector Machine	0.79	0.57	1.00	0.88	0.78	0.82	80.0	77.8	0.93
Naïve Bayes	0.64	0.39	0.89	0.83	0.56	0.67	80.0	55.6	0.74

## Discussion

Correlation

**Figure 1:**
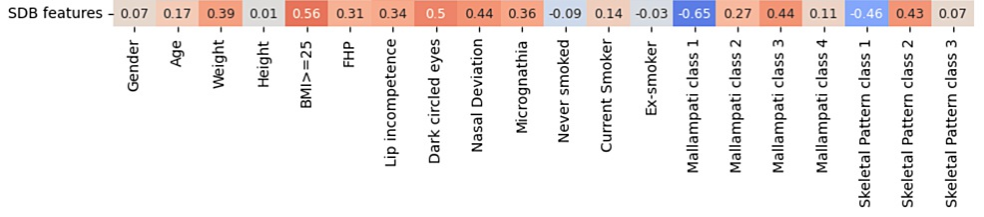
Pearson correlation coefficient (ρ) of clinical features data (bottom row) with sleep-disordered breathing (SDB) calculated by machine-learning. FHP: Forward head posture

Analysis of data produced by ML (Figure 1) revealed features with positive correlations with SDB including age, BMI, periorbital hyperchromia, nasal deviation, Mallampati class 2 or 3, skeletal pattern II (convex), micrognathia, forward head posture, and current smoking status.

Age

Our study showed age has a correlation of ρ = 0.17 with SDB. This is in agreement with the Sleep Heart Health study which found the prevalence of Apnoea-Hypopnoea Index (AHI) >= 15 increases with age in a sample of 5615 adults between 40 and 98 years old living in the community [9]. AHI is a measure of OSA severity with at least 15 events per hour is considered as moderate OSA.

Elderly individuals have a decline of pharyngeal muscle function, reduced upper airway reflex sensitivity, and narrower upper airway lumen. Their pharyngeal airway is also lengthened, and the hyoid bone is lower. All of the above contributes to increased airway resistance (ρ = 0.56; p < 0.01), tendency for airway collapse (ρ = 0.69; p < 0.01) independent of BMI and gender [10].

BMI

Obesity is well recognised as the most important risk factor for SDB. Here, we found a strong correlation of ρ = 0.51 with SDB when BMI is 25 or greater (Figure 1). The Wisconsin sleep cohort study found that a 10% increase in body weight in a person with mild SDB can produce a six-fold increase in odds of developing moderate-severe SDB [5]. Fat deposition around the neck leads to the enlargement of soft tissues around the upper neck and tongue resulting in the narrowing of the pharyngeal airway [11]. Huang and Gao (2021) concluded that obesity and abnormal craniofacial features both contribute synergistically to the narrowing of the pharyngeal airway [12].

Smoking

Although the causal link between smoking and SDB has not been fully established, the Wisconsin Sleep Cohort Study found current smokers are more likely to have SDB than those who never smoked with an adjusted odds ratio (OR) of 3.05 (95% CI 1.44-6.44). Former smokers are at a slightly higher risk of having SDB than never-smokers, OR 1.33 (95% CI 0.77-2.30). This would suggest ceasing smoking would be beneficial in treating SDB [13].

Smoking also has a dose-response relationship with SDB. Smoking 40 cigarettes or more per day has an odds ratio of 8.38 (95% CI 1.64-1.94) versus never smokers [13]. Our results showed current smokers are positively correlated with SDB with a coefficient of 0.14, whilst never smoked or ex-smokers have a negative correlation of (ρ = -0.09) and (ρ = -0.03) respectively with SDB (Figure 1).

Forward Head Posture

Some patients with Obstructive Sleep Apnoea Hypopnoea Syndrome (OSAHS) have a restricted upper airway. They compensate for this by adopting a forward head posture which extends the neck forward to open the cranio-cervical (OPT/NSL) angle, making the airway wider. Studies have found that OPT/NSL has an extremely high correlation (ρ = 0.807, p<0.01) with an increase in pharyngeal airway space (PAS) [14]. Forward head posture also helps to adopt compensatory mouth breathing in patients with nasal or nasopharyngeal obstruction. Our ML models calculated a correlation of ρ = 0.31 between FHP and SDB (Figure 1).

Lip Incompetence

Mouth breathing in children to compensate for nasal obstruction due to various pathologies such as adenoid/tonsil hypertrophy, nasal septum deviation, hypertrophic turbinates, and chronic sinusitis can result in characteristic changes in facial skeletal development persisting into adulthood. These include upper lip incompetence, lowered hyoid bone (at the level of C4-C6 vertebrae), narrow upper dental arch, increased anterior lower face height, retrognathia, and reduced nose width and prominence [15]. The correlation between lip incompetence and SDB was measured at ρ = 0.34 in our study (Figure 1).

Micrognathia, Retrognathia, Dentofacial Skeletal Pattern

Cephalometric radiographs have shown that pharyngeal airway space is influenced by craniofacial morphology. PAS is determined by the size and position of the uvula, tongue, and mandible. Longer and angulated uvula is more likely to be found in adults with mandibular retrognathism or prognathism. Their PAS is reduced compared to those with normal mandibles [16].

Patients with retrognathism, that is, Class II skeletal pattern have a downward displacement of the tongue which puts it in contact with the soft palate. When supine, the tongue pushes onto the soft palate dorsally causing obstruction. Numerous studies have demonstrated the circular relationship between retrognathia and OSA. In a recent study, Tepedino et al. (2020) confirm that mandibular length is negatively correlated (p <  0.001; ρ = −0.37) with AHI severity in the Spearman’s rho test [17].

The following craniofacial features were found to have a positive correlation with SDB in our models: micrognathia (ρ = 0.36), skeletal pattern class II (ρ = 0.43), nasal deviation (ρ = 0.44).

Periorbital Hyperchromia

The causal link between poor sleep and periorbital hyperchromia (dark-circle eyes) is not well established. Some researchers proposed that poor sleep causes oxidative stress, skin damage, inflammation, and impaired skin recovery. The skin around the eyes is particularly delicate and sensitive to any changes in microcirculation. Stress and aging can cause an increase in the permeability of vasculature resulting in local oedema, extravasation and reduced circulation of deoxygenated blood [18].

Mallampati

As expected, Mallampati class 1 and normal skeletal pattern (class I) have negative correlations with SDB in our models. Surprisingly, the most severe class of Mallampati (class 4) has only a weak correlation with SDB (ρ = 0.11). Mallampati class 3 has the highest positive correlation with SDB (ρ = 0.44).

Model performance

The classification accuracy achieved in this study ranges from 71% to 93%. Using machine learning for SDB prediction here is justifiable when comparing against the results of a machine-learning project by Holfinger et al. (2022) which used artificial neural network (ANN), random forests (RF), and SVM to predict OSA from a cohort of 17,448 subjects based on age, sex, BMI, and race. The AUCs from that study were between 0.66 and 0.68 [19]. Our research to date has not revealed any literature on the use of ML to predict SDB from predominantly craniofacial features.

The major limitation of this study is the small sample size (n=69). A larger dataset would give the models more opportunities to train and yield better results. One of the major advantages of machine learning is its ability to process a vast number of input features (independent variables). Only 12 input features were used here. It would be appropriate to consider ML models training on a large dataset which incorporates all the structural and non-structural risk factors of SDB.

Another limitation of our sampled data is race. A literature-based analysis by Benjafield et al. (2019) has found countries most affected by OSA are China, the USA, Brazil, and India [20]. A vast majority of the subjects in our study were from South-East Asia and for that reason, race was excluded from the ML dataset.

We recognise that our data sample has a gender bias with a higher percentage of female subjects. This is due to the limitation of drawing data from a restricted source, that is, a private dental clinic where female patients are more likely to seek treatment. Access to a larger database, such as from a public hospital or sleep study centre, would address this issue.

Only four basic ML models were explored here since this study aimed to assess the possibility of ML for SDB prediction as a proof of concept. With a larger and broader dataset, more sophisticated models can be used such as Decision Tree, Random Forests, Artificial Neural Networks, Deep Learning and so on. Advanced methods of data preparation and tuning such as k-fold cross validation, hyperparameter tuning can also be used to yield better performance.

## Conclusions

In this paper, Logistic Regression, Support Vector Machine, K-nearest neighbours, and Naïve Bayes machine models were used on a dataset of 69 adult subjects with varying age, BMI, and craniofacial features to predict the presence of SDB. Logistic Regression and Support Vector Machine performed well with accuracy, F1 score and AUC nearing 90%.

Traditionally, doctors are trained to use their own intuition, experience and knowledge from empirical evidence borne out of standard statistical methods to predict a diagnosis. A well-developed, robust machine learning algorithm has the potential to be an adjunct or screening tool for the presence of SDB in a patient and thereby, assist the clinician on planning the next step in management.
